# Interactions of *Aspergillus fumigatus* and *Stenotrophomonas maltophilia* in an *in vitro* Mixed Biofilm Model: Does the Strain Matter?

**DOI:** 10.3389/fmicb.2018.02850

**Published:** 2018-11-27

**Authors:** Elise Melloul, Lolita Roisin, Marie-Fleur Durieux, Paul-Louis Woerther, Delphine Jenot, Veronica Risco, Jacques Guillot, Eric Dannaoui, Jean-Winoc Decousser, Françoise Botterel

**Affiliations:** ^1^EA 7380 Dynamyc, Ecole Nationale Vétérinaire d’Alfort, Université Paris-Est Créteil, Créteil, France; ^2^Service de Parasitologie-Mycologie, Limoges, France; ^3^Unité de Bactériologie-Hygiéne, Département de Microbiologie, Assistance Publique – Hôpitaux de Paris, Hôpital Henri Mondor, Créteil, France; ^4^Unité de Parasitologie-Mycologie, Ecole Nationale Vétérinaire d’Alfort, Maisons-Alfort, France; ^5^Unité de Parasitologie-Mycologie, Service de Microbiologie, Hôpital Européen Georges Pompidou, Assistance Publique – Hôpitaux de Paris, Faculté de Médecine, Université Paris-Descartes, Paris, France; ^6^Unité de Parasitologie-Mycologie, Département de Microbiologie, Groupe Hospitalier Henri Mondor – Albert Chenevier, Assistance Publique – Hôpitaux de Paris, Université Paris-Est Créteil, Créteil, France

**Keywords:** bacterial-fungal interactions, *Aspergillus fumigatus*, Stenotrophomonas maltophilia, mixed biofilm, antibiosis, *Galleria mellonella*

## Abstract

**Introduction:** *Aspergillus fumigatus* (Af) and *Stenotrophomonas maltophilia* (Sm) are pathogenic microorganisms, which coexist in the respiratory tract of cystic fibrosis (CF) patients. We recently developed an *in vitro* model of mixed biofilm associating Af ATCC 13073-GFP (Af13073) and Sm ATCC 13637 (Sm13637) and described an antibiosis effect. The present study aim was to assess the antibiosis of Sm on Af using different strains and to analyze the potential synergistic virulence of these strains in an *in vivo Galleria mellonella* model.

**Methods:** The effect of Sm13637 was evaluated on eight Af strains and the effect of nine Sm strains was evaluated on Af13073. The strains originated from clinical cases (human and animal) and from environment. Fungal and bacterial inocula were simultaneously inoculated to initiate mixed biofilm formation. Fungal growth inhibition was analyzed by qPCR and CLSM and the fungal cell wall modifications by TEM analysis. The virulence of different Sm strains was assessed in association with Af in *G. mellonella* larvae.

**Results:** All strains of Af and Sm were able to produce single and mixed biofilms. The antibiosis effect of Sm13637 was similar whatever the Af strain tested. On the other hand, the antibiosis effect of Sm strains was bacterial-fitness and strain dependent. One strain (1/9) originated from animal clinical case was never able to induce an antibiosis, even with high bacterial concentration. In the *G. mellonella* model, co-inoculation with Sm13637 and Af13073 showed synergism since the mortality was 50%, i.e., more than the summed virulence of both.

**Conclusion:** Human clinical strains of Sm yielded in higher antibiosis effect on Af and in a thinner mixed biofilm, probably due to an adaptive effect of these strains. Further research covering Af increased wall thickness in the presence of Sm strains, and its correlation with modified antifungal susceptibility is encouraged in patients with chronic respiratory infections by these 2 microorganisms.

## Introduction

Polymicrobial biofilm communities are commonly found in lungs of cystic fibrosis (CF) patients or in the respiratory tract of patients with chronic lung diseases (Frey-Klett et al., 2011). In biofilms, microorganisms are enclosed in an extracellular matrix (ECM) of self-produced extracellular polymeric substance that confers a protective and antimicrobial-resistant environment (Donlan and Costerton, 2002; Frey-Klett et al., 2011). From the progressive understanding of the complexities of polymicrobial biofilm, it is becoming all the more evident that interactions between bacterial and fungal pathogens can influence pathogenesis and antimicrobial resistance (Peters et al., 2012; Baldan et al., 2014; Beaume et al., 2015). However, it is often difficult to elucidate whether these clinical changes are a cause or a consequence of these interactions (O’Brien and Fothergill, 2017). Among microorganisms described in mixed biofilms of the respiratory tract of CF patients, *Aspergillus fumigatus* (Af) (Fischer et al., 2014; Williams et al., 2016), a filamentous fungus, and *Pseudomonas aeruginosa* (Baxter et al., 2013; Reece et al., 2017), a Gram-negative bacillus are the most frequently documented. Several studies have reported *A. fumigatus* colonization in patients with chronic lung disease, especially chronic obstructive pulmonary disease (COPD) and CF, probably due to the lung structural changes and the heavy courses of antibiotics (Bafadhel et al., 2014; Sabino et al., 2015). *Stenotrophomonas maltophilia* (Sm) is an emergent ubiquitous Gram-negative bacillus (Chotirmall and McElvaney, 2014) whose antibiotic resistance and biofilm formation capacity promote opportunistic pathogenicity in CF or in immunocompromised patients (Calza et al., 2003; Jacquier et al., 2011; Cabaret et al., 2016). Recently, *S. maltophilia* was positively associated with *Aspergillus* species infections in patients with CF suggesting that such co-infections are common and may alter therapeutic responses in these patients (Granchelli et al., 2018). Many studies have examined the interactions between pathogens isolated in CF patients: Pompilio et al. (2015) showed that *P. aeruginosa* and *S. maltophilia* have reciprocal interferences and *S. maltophilia* could modulate the *P. aeruginosa* virulence in CF patients. In the same way, some studies revealed antagonism or synergism effects of *P. aeruginosa* on *A. fumigatus* (Briard et al., 2015, 2016, 2017; Anand et al., 2017). For our work and in order to better understand the interactions between *A. fumigatus* and *S. maltophilia* and to evaluate the feasibility of developing their mixed biofilm in the respiratory tract, we previously developed an *in vitro* model of such mixed biofilm (Melloul et al., 2016). Analytical and quantitative methods revealed typical structures of biofilm with production of an ECM enclosing fungal hyphae and bacteria. We also demonstrated the antibiosis effect of *S. maltophilia* on *A. fumigatus*, i.e., the fungal hyphae were highly branched and abortive, the fungal cell wall was thicker and its growth was inhibited (Melloul et al., 2016). Similar antibiosis effect has already been reported on different *A. fumigatus* strains in contact with *P. aeruginosa* or *S. aureus* (Briard et al., 2015; Ramirez Granillo et al., 2015). In our previous study, two clinical strains were chosen: *S. maltophilia* ATCC 13637 (Sm13637), isolated from mouth-cancer patient, and *A. fumigatus* ATCC 13073 (Af13073) isolated from an invasive aspergillosis case.

However, the mechanisms underlying the pathogenesis and the virulence of different strains of *A. fumigatus* and of *S. maltophilia*, remain poorly investigated. No information is available regarding the correlation between the formation of these mixed biofilms and pathogenicity. The aim of the present study was to assess the effect of associating different fungal and bacterial strains on the antibiosis effect and the virulence of these associations *in vivo* in *Galleria mellonella* model. *A. fumigatus* and *S. maltophilia* are ubiquitous species and some environmental strains could colonize the respiratory tract and be in contact with clinical strains (Loeffert et al., 2018). Thus, it seems interesting to evaluate the antibiosis phenomenon in presence of strains from diverse origins. Different fungal strains originated from diverse origins (human or animal clinical cases or environment) have been tested in association with a bacterial reference strain (human clinical case) and vice versa.

## Materials and Methods

### Ethics Statement

The authors testify that all procedures contributing to this work are in compliance with the ethical standards of the “Réglementation et éthique de l’expérimentation animale” and with the Helsinki Declaration of 1975, as revised in 2008. Since the study was retrospectively conducted on isolates collected through routine clinical work and patient’s identifiable information had already been anonymized, no written or verbal informed consent was necessary for patients to participate in this study. Additionally, the authors had no contact or interaction with the patients.

### Microbial Strains and Inocula

*A. fumigatus* ATCC 13073-GFP (Af13073), provided by Wasylnka and Moore (2002), and *S. maltophilia* ATCC 13637 (Sm13637) strains were used in the previous study to develop the model of mixed biofilm (Melloul et al., 2016). In this work, nine strains of *S. maltophilia* (including Sm13637) were selected to create mixed biofilm with Af13073, and eight *A. fumigatus* strains (including Af13073) were used with Sm13637 (Table 1). The *A. fumigatus* and *S. maltophilia* strains originated from human, animal and environment were stored at -20°C in glycerol and, respectively, streaked out on 2% malt agar (+ 0.05% chloramphenicol) for 7 days at 37°C and on CHO-plate (Columbia agar + 5% horse blood) (BioMérieux, Marcy-l’Etoile, France) for 24 h at 37°C.

**Table 1:** Origin of the *A. fumigatus* and *S. maltophilia* strains tested.

Species	Strain name	Origin	Samples	Genogroups
*S. maltophilia*	Sm13637 (ATCC)	Human	Mouth (oral carcinoma)	6
*S. maltophilia*	Sm_6-1	Human	Sputum (CF)	6
*S. maltophilia*	Sm_6-2	Human	Deep Catheter	6
*S. maltophilia*	Sm_6-3	Animal (Dog)	Transtracheal aspiration	6
*S. maltophilia*	Sm_2-1	Animal (Horse)	Transtracheal aspiration	2
*S. maltophilia*	Sm_2-2	Animal (Horse)	Transtracheal aspiration	2
*S. maltophilia*	Sm_D-1	Hospital environment	Endoscope	D
*S. maltophilia*	Sm_D-2	Hospital environment	Endoscope	D
*S. maltophilia*	Sm_D-3	Hospital environment	Gastroscope	D
*A. fumigatus*	Af13073 (ATCC)	Human	Bronchoalveolar lavage	NA
*A. fumigatus*	Af_H1	Human	Bronchoalveolar lavage	NA
*A. fumigatus*	Af_H2	Human	Bronchoalveolar lavage	NA
*A. fumigatus*	Af_A1	Animal (Horse)	Guttural pouch	NA
*A. fumigatus*	Af_A2	Animal (Duck)	Bronchoalveolar lavage	NA
*A. fumigatus*	Af_E1	Environment	Soil	NA
*A. fumigatus*	Af_E2	Environment	Soil	NA
*A. fumigatus*	Af_E3	Environment	Soil	NA

*NA, not available.*

All *A. fumigatus* strains were confirmed by molecular identification, which was performed by sequencing part of beta-tubulin and calmodulin genes. DNA sequences were analyzed using Seqscape v2.5 (Applied Biosystems, Villebon Sur Yvette, France) and were compared using Genbank and Mycobank databases sequences of strains belonging to *Aspergillus* section *Fumigati* (Loeffert et al., 2017). All of the eight fungal strains were identified as *A. fumigatus sensu stricto*.

All of the *S. maltophilia* strains were identified using MALDI-TOF mass spectrometry (Andromas, Beckmann Coulter, Villepinte, France). A genogrouping affiliation using MLST (MultiLocus Sequence Typing) profiles was performed. After DNA extraction using QIAsymphony DSP Mini Kit (Qiagen, Courtaboeuf, France), the MLST profile was determined using Kaiser Scheme. Briefly, seven housekeeping genes were amplified and sequenced according to PubMLST website recommendations^1^. For each strain, the seven allelic sequences of the seven housekeeping genes were concatenated and aligned using Seaview 4.0^2^ (Corlouer et al., 2017).

The inocula were prepared as described in our previous study protocol (Melloul et al., 2016) and adjusted in MOPS [3 (N-morpholino) – propanesulphonic acid – buffered RPMI (Roswell Park Memorial institute) 1640 [pH 7.0] (Sigma-Aldrich, Saint-Quentin Fallavier, France) + 10% of FBS (foetal bovine serum) (Sigma-Aldrich, Saint-Quentin Fallavier, France) to obtain the final working concentrations for each experiment.

### Single and Mixed Biofilm Formation

The preparation steps for mixed biofilm formation were prepared as previously described (Melloul et al., 2016). Briefly, equal volumes of microbial suspensions (10^5^ conidia/mL for *A. fumigatus* and 10^6^ cells/mL for *S. maltophilia*) were simultaneously added on polystyrene supports, 96-well microtiter plates (Thermo Fisher Scientific Inc., Villebon sur Yvette, France) or Lab-Tek^TM^ slides (NuncTM, Thermo Fisher Scientific Inc.). For single biofilms (Af or Sm), a volume of RPMI + FBS medium was added to microbial suspension. Biofilms were incubated at 37°C in static condition. Then, cultures were washed twice with phosphate buffer saline (PBS) to eliminate planktonic cells. To evaluate the strain-specific antibiosis effect of bacteria on fungal growth, DNA quantification of bacteria or fungi was performed on the fungal and the mixed biofilms after 16 h of culture, the time at which the fungal inhibition reached its maximum in our reference model (Melloul et al., 2016). To observe the *A. fumigatus* hyphal phenotype in the presence or absence of bacteria strains, biofilms were incubated for 24 h (Melloul et al., 2016).

### Assessment of Conidial and Bacterial Equivalent by Real Time PCR

The biofilms were washed twice before incubation for 2 h at 56°C with 250 μL of tissue lysis buffer (Buffer ATL, Qiagen). They were then scraped to recover all adherent organisms, and washed with 250 μL of PBS. The samples were then homogenized as previously described (Melloul et al., 2014) and extracted with the QIAamp DNA Mini Kit (Qiagen) according to the manufacturer’s instructions. Quantification of the amount of *A. fumigatus* and *S. maltophilia* DNA was performed by multiplex qPCR targeting 28S rRNA and 23S rRNA genes, respectively, following the protocol already described in our previous study (Melloul et al., 2016). Fluorescence curves were analyzed using LightCycler software V3.5 and results were expressed in conidial equivalent (CE) or bacterial equivalent (BE) in comparison with a standard curve plotted on DNA samples extracted from co-inoculated solutions with different doses of *A. fumigatus* conidia (1–10^8^ conidia) and *S. maltophilia* (10–10^9^ cells). This experiment was performed in duplicate using three samples per biofilm.

### Confocal Laser Scanning Microscopy (CLSM)

Investigations of *A. fumigatus* phenotypic modifications in mixed biofilm, as compared with those seen in fungal biofilm, were carried out by CLSM after 24 h of culture at 37°C. Af13073-GFP was visualized with FITC filter. The others *A. fumigatus* strains were visualized after Calcofluor-white (Invitrogen, Villebon sur Yvette, France) coloration with DAPI filter. The biofilms were examined under Zeiss LSM 510 META microscope (Zeiss, Oberkochen, Germany). CLSM was used to measure biofilm thicknesses, via Z stack analyses, using ImageJ program^3^.

### Transmission Electron Microscopy (TEM)

The biofilm preparation for TEM analyses was performed as previously described (Melloul et al., 2016). Briefly, samples were fixed with 2.5% glutaraldehyde-cacodylate buffer (pH 6.5) and then post-fixed with osmium tetroxide and dehydrated with different dilutions of alcohol (50–100%). The samples were then embedded into EPON resin and left to polymerize. Ultra-fine sections were obtained using a Leica UC7-RT ultramicrotome, and contrasted with lead-citrate and uranyl-acetate solutions. Specimens were mounted on grids to be examined under the microscope (JEOL 100 CX II instrument, Japan). TEM was used to measure the cell wall thickness of *A. fumigatus* in single and mixed biofilms using ImageJ program. For both biofilms, between 10 and 20 measurements of cell wall thickness were performed on 15 different hyphae.

### Growth Phenotype of *S. maltophilia* Strains

*S. maltophilia* inocula at 10^8^ cells/mL were incubated at 25 or 37°C with shaking (200 rpm: planktonic phase) for 24 h or under static condition (biofilm phase) for 4 h (adhesion assay) or 24 h (biofilm formation assay). The analysis of planktonic bacterial growth was performed by spectrophotometry. The absorbance was measured relative to medium alone at 600 nm by optical density. The adhesion and biofilm formation assays were assessed by crystal violet staining method, as previously described (Melloul et al., 2016). After 4 or 24 h of culture in static condition, the samples were washed twice with PBS. Then, a solution of 200 μL of crystal violet 0.1% was added to wells and samples were incubated for 20 min at room temperature and washed three times with PBS before adding 200 μL of acetic acid (30%). These analyses were performed on the spectrophotometer at 550 nm (MultiskanTM FC, Thermo Fisher Scientific Inc.).

### Co-infection of *A. fumigatus* and *S. maltophilia* in *Galleria mellonella* Model

An *in vivo* infection model with *A. fumigatus* and *S. maltophilia* was constructed using larvae of *G. mellonella* to determine the virulence of these two pathogens separated and together. The reference strain Af13073 was tested alone and in co-inoculation with six different *S. maltophilia* strains (Sm13637, Sm_6-1, Sm_2-1, Sm_2-2, Sm_D-1, and Sm_D-2). *A. fumigatus* inoculum of 10^7^ conidia/mL and *S. maltophilia* inocula of 10^8^ CFU/mL each were prepared in PBT-Tween (PBST). Survival curves were drawn after single inoculation of 10 μL of bacterial or conidial solution in the last left pro-leg using a Hamilton syringe. For co-inoculation, 20 μL of mixed solution of *A. fumigatus* and *S. maltophilia* was injected. A group of non-inoculated larvae and a group of larvae inoculated with 20 μL PBST were used as controls. The larvae were incubated in Petri dishes at 37°C for 7 days and their mortality, defined by absence of movement after touch, was daily checked. The results from three separate experiments were combined.

### Statistical Analysis

Statistical analyses were performed using JMP 13.0 software. Data of continuous variables are presented in means ± standard errors of the mean. *P*-value was significant if <0.05. Data were analyzed by Kruskal-Wallis (one-way variance analysis) test in order to compare the concentrations of *A. fumigatus* and/or *S. maltophilia* in single and mixed biofilms. Linear regression and correlation (Spearman rank correlation) were conducted to assess the relationship between the concentration of bacteria and fungi in mixed biofilms. Microscopy data (TEM and CLSM) were subjected to Wilcoxon and Kruskal–Wallis tests to compare the means of biofilm and fungal cell wall thicknesses in the single and mixed biofilms. The virulence data were analyzed by Kaplan–Meier survival curves using JMP 13.0 software. The different groups were compared to negative control and between them using log-rank test (JMP 13.0).

## Results

### Genotyping of *S. maltophilia* Strains

The analysis and the comparison of genome sequencing of the nine S. maltophilia strains showed three different genogroups (6, 2, and D) (Table 1). The two clinical strains isolated from humans and the one isolated from a dog were of genogroup 6 and named Sm_6-1, Sm_6-2, and Sm_6-3, respectively. The reference strain Sm13637 also belonged to genogroup 6. The strains isolated from horse trachea belonged to genogroup 2 and named Sm_2-1 and Sm_2-2. The strains originated from hospital environment belonged to genogroup D and named Sm_D-1, Sm_D-2, and Sm_D-3. These isolates were not genetically related.

### Phenotype Modifications of Different *A. fumigatus* Strains in the Presence of Sm13637

**FIGURE 1:**
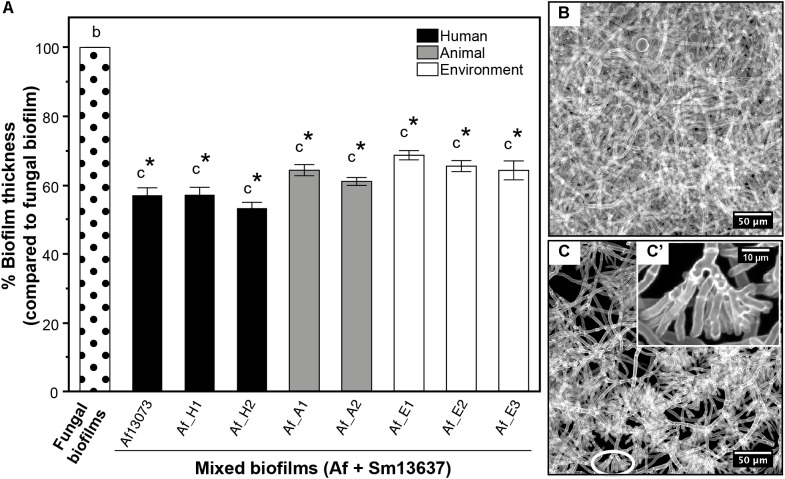
*A. fumigatus* phenotype and thickness of single and mixed biofilms by fungal origin. **(A)** Percent of mixed biofilms thickness (compared with single fungal biofilms) by *A. fumigatus* origin: human (black), animal (gray) and environment (white). **(B)** Example of single fungal biofilm of Af_H1. **(C)** Example of mixed biofilm of Af_H1 and Sm13637. **(C’)** Zoom of shorter and atrophied ramifications and highly branched hyphae (white circles). The letters indicate the phenotype observed: b, photo B; c, photo C. ^∗^*p* < 0.05 compared with single fungal biofilms; Af, *A. fumigatus.*

Seven combinations of *S. maltophilia* and *A. fumigatus* strains were tested *in vitro* according to the mixed biofilm model protocol and in comparison with our reference model (Af13073 + Sm13637). Each of the different *A. fumigatus* strains was co-inoculated at a concentration of 10^5^ conidia/mL with Sm13637 at 10^6^ cells/mL. Upon comparison with the single fungal biofilms, the morphological aspect of the seven *A. fumigatus* strains in the mixed biofilms was modified in the presence of Sm13637 (Figure 1), i.e., exhibiting atrophied structures and highly branched patterns with shorter ramifications at the tip, as previously described with Af13073 and Sm13637. The CLSM analysis showed a significant decrease of mixed biofilms thicknesses compared with fungal biofilms, irrespective of *A. fumigatus* strains origin (*p* < 0.0001) (Figure 1A). The mean thicknesses of single fungal biofilms were 48.1 ± 1.1, 38.9 ± 1.0, and 30.3 ± 0.8 μm for *A. fumigatus* strains originated from human, animal and environment, respectively, with no significant difference between these strains and Af13073. The thickness of mixed biofilms was divided by 1.8, 1.6, or 1.5 (mean of 27.3 ± 0.7, 24.5 ± 0.7, and 20.5 ± 0.6 μm) for *A. fumigatus* strains originated from humans, animals and environment, respectively, compared with single fungal biofilms with no difference between the strains themselves (Figure 1A).

The quantification of fungal DNA (log CE/mL) was run for the seven *A. fumigatus* strains in the single and mixed biofilms with Sm13637, and compared with the reference strain Af13073. Sm13637 strain significantly reduced the fungal growth in all mixed biofilms tested with the eight *A. fumigatus* strains (*p* < 0.0001) (Table 2). The difference of fungal DNA concentrations between fungal and mixed biofilms, for each tested *A. fumigatus* strain was comprised between 0.8 and 1.2 log. There was no significant difference between the strains and the reference Af13073 in this concern.

**Table 2:** Quantification of fungal DNA in single and mixed biofilms by Af strains.

Strains	Log CE/mL ± SE		
	Fungal biofilm	Mixed biofilm (with Sm13637)	*P*-value
Af13073	7. 10 ± 0.02	5.97 ± 0.06	<0.0001 ^∗^
Af_H1	7.59 ± 0.05	6.69 ± 0.21	0.0051 ^∗^
Af_H2	7.62 ± 0.02	6.78 ± 0.16	0.0051 ^∗^
Af_A1	7.63 ± 0.02	6.49 ± 0.12	0.0051 ^∗^
Af_A2	7.17 ± 0.06	5.91 ± 0.19	0.0003 ^∗^
Af_E1	7.15 ± 0.03	6.23 ± 0.03	0.0051 ^∗^
Af_E2	6.80 ± 0.03	5.62 ± 0.13	0.0051 ^∗^
Af_E3	6.76 ± 0.06	5.84 ± 0.08	0.0051 ^∗^

*Log CE (fungi)/mL ± standard error, ^∗^p-value is significant if <0.05, Af, A. fumigatus; Sm, S. maltophilia.*

**FIGURE 2:**
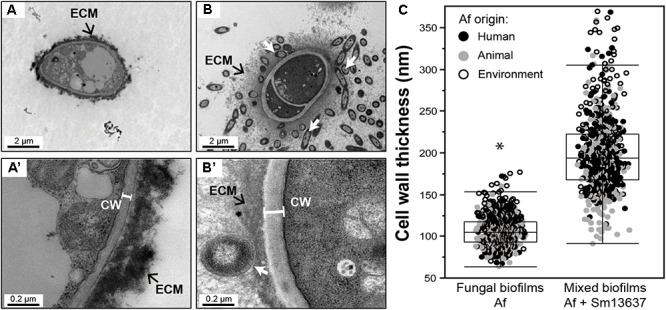
Modification and measurement of cell wall thickness of *A. fumigatus* in single and mixed biofilms, by fungal strains. **(A,A’)** Single fungal biofilm. **(B,B’)** Mixed biofilm with Sm13637 bacteria (white arrows). **(A’,B’)** Zoom of fungal cell wall. **(C)** TEM, measurement of fungal cell wall thickness by *A. fumigatus* origin: human (black), animal (gray) and environment (white). ECM, extracellular matrix; CW, cell wall. ^∗^*p* < 0.05 compared with single fungal biofilms; Af, *A. fumigatus.*

As already observed with Af13073 in our previous study, a mixed biofilm was created with each of the *A. fumigatus* strains, as evidenced by the presence of the extracellular matrix (ECM) (Figure 2). The cell wall thickness of *A. fumigatus* hyphae of strains originated from humans, animals and environment was modified in the presence of Sm13637, in a similar way to what was observed with Af13073 (Figures 2A,B), i.e., the fungal cell wall thickness significantly increased (*p* < 0.0001). This result was supported by the highly branched phenotype observed via CLSM analysis (Figure 1).

### Antibiosis Effect of *S. maltophilia* Strains on Af13073

**FIGURE 3:**
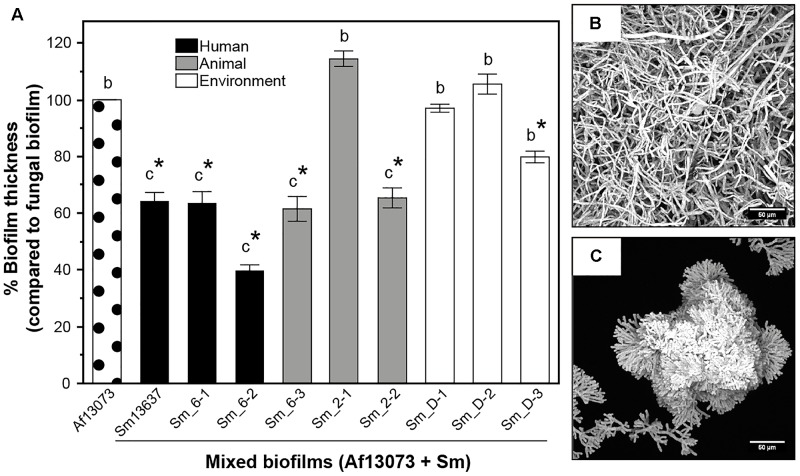
Reduction of mixed biofilms thicknesses and modification of Af13073 phenotype according to *S. maltophilia* strains. **(A)** Thicknesses of mixed biofilms compared with thickness of Af13073 vs. *S. maltophilia* strains: human (black), animal (gray) and environment (white). **(B)** Wild type phenotype of Af13073 in the presence of *S. maltophilia* **(C)** Modified phenotype of Af13073 in the presence of *S. maltophilia*. The letters indicate the phenotype observed: b, photo B; c, photo C. ^∗^*p* < 0.05 compared with single fungal biofilm; Sm, *S. maltophilia.*

Eight combinations of Af13073 with different *S. maltophilia* strains were tested *in vitro* and compared with the reference model (Sm13637 + Af13073). As previously observed with Sm13637, a mixed biofilm was created with all *S. maltophilia* strains, as evidenced by the presence of the extracellular matrix surrounding pathogens (data not shown). However, at 37°C, the bacterial antibiosis effect on Af13073 was not observed with all of the *S. maltophilia* strains. After 24 h of culture, the thickness of mixed biofilms was less than that of fungal biofilm when Af13073 was in co-culture with *S. maltophilia* strains of genogroup 6 (Sm13637, Sm_6-1, Sm_6-2, Sm_6-3), of genogroup 2 (only one, Sm_2-2) and of the environmental strain Sm_D-3 (Figure 3A). Compared with Sm_D-3 that presented wild type phenotype (Figures 3A,B), strains of genogroup 6 (Sm13637, Sm_6-1, Sm_6-2, Sm_6-3) and Sm_2-2 induced shorter ramifications, highly branched patterns and a decrease of biofilm thickness (Figures 3A,C). In contrast, no hyphae modification or biofilm thickness decrease was induced by strain Sm_2-1 and other strains of genogroups D (Figure 3).

**FIGURE 4:**
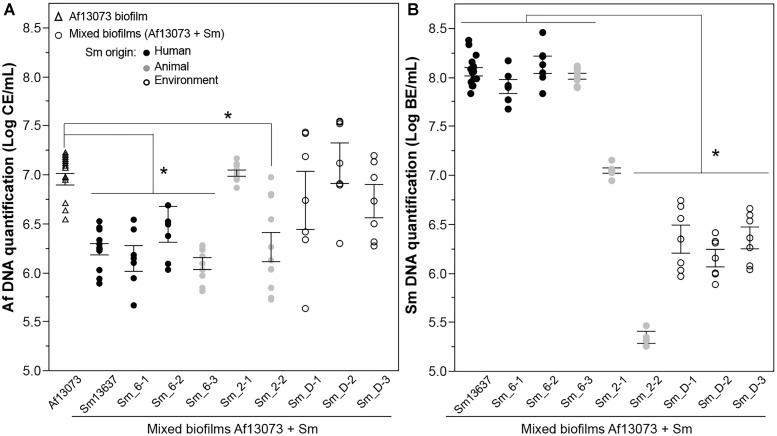
Quantification of fungal and bacterial DNA in mixed biofilms by *S. maltophilia* strains. **(A)** Quantification by qPCR of Af13073 (Log CE/mL) in single and mixed biofilms with Sm strains from human (black), animal (gray), and environment (white) (at 10^6^ cells/mL). **(B)** Quantification by qPCR of Sm (Log BE/mL) in the mixed biofilms (Af13073 + Sm at 10^6^ cells/mL); ^∗^*p* < 0.05; Af, *A. fumigatus*; Sm, *S. maltophilia*.

The quantification of bacterial and fungal DNA (log BE or CE/mL, respectively) was also performed for the eight *S. maltophilia* strains Sm13637 and Af13073 in the mixed biofilms, and compared with the fungal DNA in the single biofilm (Af13073). In the mixed biofilms, the quantification of fungal DNA showed a significant inhibition of fungal growth in the presence of clinical *S. maltophilia* strains of genogroup 6 (*p* < 0.0001) (Figure 4A). The concentration of bacterial DNA in these biofilms was higher than 10^8^ BE/mL (Figure 4B). Interestingly, Sm_2-2 strain (animal, genogroup 2) induced an inhibition of *A. fumigatus* growth (*p* = 0.0104), but the bacterial concentration in the mixed biofilm was low, below 10^5^ BE/mL (Figure 4). In contrast, mixed biofilm associating Af13073 and *S. maltophilia* strains from genogroup D (environment) or Sm_2-1 (animal, genogroup 2) did not exhibit a significant inhibition of fungal DNA concentration (*p* = 0.419) compared with single biofilm of Af13073 (Figure 4A). The bacterial DNA concentration in mixed biofilm was low for the environmental strains of *S. maltophilia* (average of 10^6^ BE/mL), but relatively high for the Sm_2-1 strain originated from animal (10^7^ BE/mL) (Figure 4B). The concentration of *S. maltophilia*-genogroup 6 DNA in the mixed biofilm was higher than that of genogroup D (*p* < 0.001). The bacterial and fungal concentrations were inversely related (Spearman’s ρ = -0.4572, *p* = 0.0075). When the concentration of bacteria was high, the concentration of *A. fumigatus* was low and vice versa. Sm_2-1 and Sm_2-2 strains of genogroup 2 did not behave in the same way. Sm_2-1 developed properly in the mixed biofilm but had no impact on the fungal DNA concentration. Conversely, Sm_2-2 strain induced a decrease of fungal biomass (*p* < 0.0001) despite its weaker bacterial growth compared with Sm13637 (*p* < 0.0386) (Figure 4).

The highly branched fungal phenotype observed in the presence of *S. maltophilia* strains of genogroup 6 was always correlated with an increase of more than twofold in the fungal cell wall thickness (data not shown).

### Growth of *S. maltophilia* Strains in Planktonic or Biofilm Conditions According to Temperature Level

**FIGURE 5:**
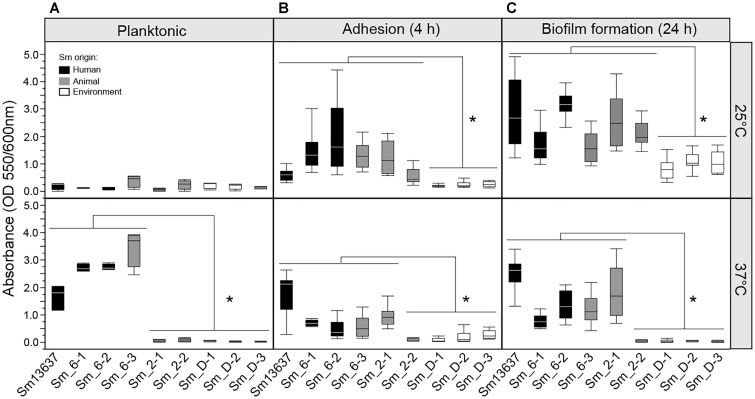
Growth, adhesion, and biofilm formation of *S. maltophilia* strains by temperature degree. Culture of *S. maltophilia* strains originated from human (black), animal (gray), and environment (white), in RPMI + FBS media at 25 and 37°C. **(A)** Planktonic bacterial growth measured by absorbance (OD 600 nm) after 24 h of culture. **(B)** Bacterial adhesion on polystyrene support after 4 h measured by crystal violet (OD 550 nm). **(C)** Biofilm formation after 24 h of culture measured by crystal violet (OD 550 nm); ^∗^*p* < 0.05.

The growth and fitness of different *S. maltophilia* strains were evaluated at 25 and 37°C in RPMI + FBS media to analyze the impact of the temperature factor on the mixed biofilm model. Temperature had a positive effect on planktonic growth of *S. maltophilia* strains of genogroup 6 (*p* < 0.0001) (Figure 5A). Indeed, these strains grew faster at 37°C than at 25°C. Conversely, the strains of genogroups 2 and D grew equally at 37 and 25°C (Figure 5A). All of the tested bacteria were able to adhere to polystyrene surface after 4 h of culture in static condition, regardless of temperature (Figure 5B). However, the adhesion was weaker for the three strains of genogroup D and one strain of genogroup 2 (Sm_2-2) compared with the other strains. The results of adhesion were correlated with the formation of biofilm (after 24 h of culture). Thus, strains of genogroup 6 and Sm_2-1 of genogroup 2 were able to form the strongest biofilms (*p* < 0.0001) (Figure 5C).

**FIGURE 6:**
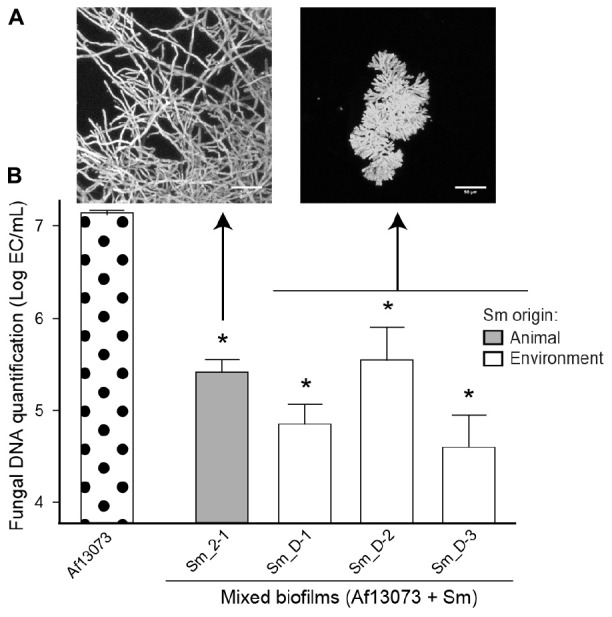
Dose-dependent fungal biomass inhibition in mixed biofilms. **(A)** Af13073 phenotype in mixed biofilm with *S. maltophilia* strains from animal (gray) or environment (white). **(B)** Quantification by qPCR of Af13073 in fungal and mixed biofilms with 10^8^ bacteria/mL; ^∗^*p* < 0.001 compared with Af13073 biofilm; Af, *A. fumigatus*, Sm, *S. maltophilia.*

Bacterial strains of genogroup 6 were able to grow at 37°C and to induce a significant decrease of fungal growth (*p* < 0.001) and to modify the phenotype of *A. fumigatus* hyphae in the presence of 10^6^ bacteria/mL. Strains of genogroup D had weaker growth at 37°C and did not modify the fungal phenotype. To the contrary to genogroup D strains, Sm_2-2 modified the fungal phenotype and decreased its biomass in spite of the low concentrations of the bacteria. Interestingly, Sm_2-1 from genogroup 2 grew at 37°C in biofilm condition but did not induce an inhibition of growth or a modification of *A. fumigatus* phenotype at this concentration of inoculum. Consequently and in order to assess the bacterial concentration effect on its antibiosis observed on *A. fumigatus*, the bacterial inoculum was increased 100-fold (10^8^ cells/mL) for Sm_2-1, Sm_D-1, Sm_D-2, and Sm_D-3. At this bacterial concentration, the modified fungal phenotype (Figure 6A) and the reduction of the fungal growth (Figure 6B) were observed in the mixed biofilm of Af13073 and Sm_D-1, Sm_D-2, and Sm_D-3 (*p* < 0.001). In contrast, even with higher concentration (10^8^ or 10^9^ cells/mL), Sm_2-1 had no effect hyphal phenotype of Af13073, but only on the fungal growth (*p* = 0.0018) (Figure 6).

### Virulence of *A. fumigatus* and *S. maltophilia in vivo* in *G. mellonella*

**FIGURE 7:**
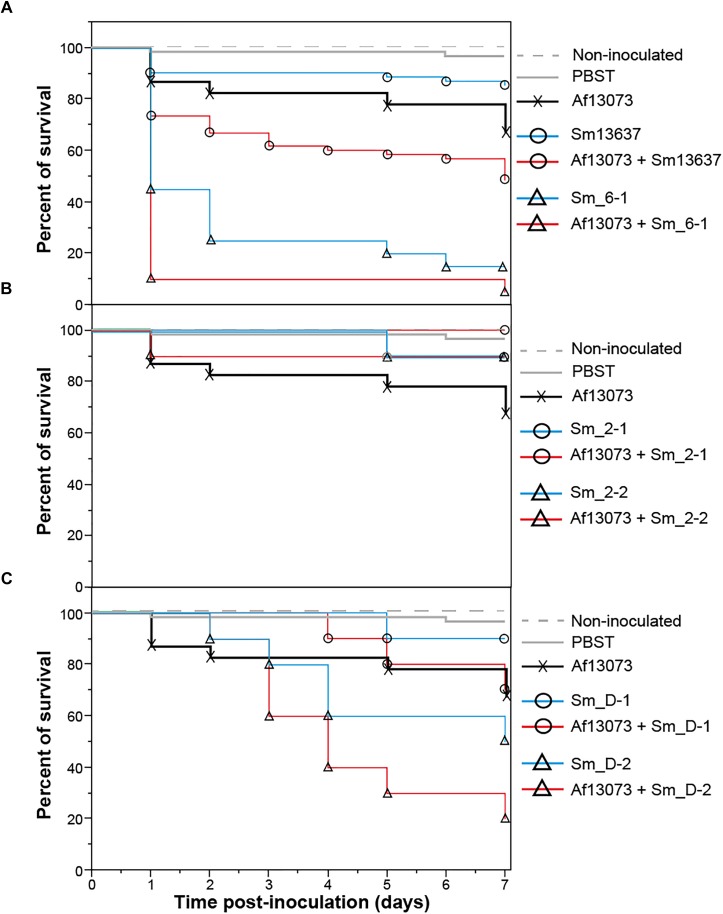
Survival curves after single and co-inoculation in *G. mellonella*. Inoculation of ten larvae per group with *S. maltophilia* strain (blue line), Af13073 (black line), or both of them (red line) depending on *S. maltophilia* strains. **(A)** Co-inoculation of Sm strains of genogroup 6 (human and animal clinical strains). **(B)** Co-inoculation of Sm strains of genogroup 2 (animal clinical strains). **(C)** Co-inoculation of Sm strains of genogroup D (environmental strains); Af, *A. fumigatus*; Sm, *S. maltophilia*.

The pathogenicity of six different strains of *S. maltophilia* was evaluated both with the bacteria alone and in association with the reference strain Af13073 in an *in vivo* model of *G. mellonella*. Two strains originated from human (Sm13637 and Sm_6-1), two from animal (Sm_2-1 and Sm_2-2) and two from the environment (Sm_D-1 and Sm_D-2) were tested *in vivo*. The control groups of larvae presented mortality of less than 5%. Af13073 strain exhibited 30% mortality rate with a concentration of 10^5^ of inoculated conidia per larvae (Figure 7). The mortality rate with *S. maltophilia* varied between the strains irrespective of the strain origin. The clinical strain isolated from a CF patient (Sm_6-1) showed more than 50% mortality in *G. mellonella* at D1 after inoculation, unlike other strains, ranging between 0 and 10% (Figure 7). Sm13637 in co-culture with Af13073 showed a synergistic effect in *G. mellonella*. Their co-inoculation resulted in 50% mortality, which was more than the sum of Sm13637 virulence (10% mortality) and Af13073 (30% mortality) (Figure 7). Sm_6-1 showed also an increase of mortality of *G. mellonella* in the presence of Af13073, with a significant synergistic effect of co-inoculation observed at D1 (90% of mortality for co-inoculation vs. 50% for Sm_6-1 and 10% for Af13073), but this mortality rate was too high to observe potential synergistic effect at D7. For *S. maltophilia* strains of genogroup 2, the survival curves observed with the bacteria alone and after co-inoculation of Af13073 and *S. maltophilia* were similar (Figure 7). Af13073 exhibited slightly higher mortality alone (30%) than in co-inoculation with Sm_2-1 or Sm_2-2 (0 and 10%) (Figure 7). For Sm_D-1, the virulence of the co-inoculation was similar to the Af13073 virulence and the presence of the bacteria did not seem to increase it. Sm_D-2, in co-inoculation with Af13073, induced a higher mortality rate (80%) at D7 compared to bacteria and fungi alone (50 and 30%, respectively) with no synergistic effect. Finally, a synergistic effect was observed with only one human clinical *S. maltophilia* strain in co-inoculation with Af13073.

## Discussion

The association of bacteria and fungi in biofilm is a common finding in lungs of CF patients or in the respiratory tract of patients with chronic lung diseases (Frey-Klett et al., 2011). In these biofilms, fungi, and bacteria have to compete or cooperate for space and nutrients and their interactions are mediated by different direct or indirect mechanisms with different degrees of specificity (spanning along the mutualism-antagonism continuum) (Hacquard, 2017). For the first time, our study demonstrated that human, animal and environmental strains of *A. fumigatus* were subjected to an antibiosis effect by *S. maltophilia*, i.e., highly branched hyphae, fungal growth inhibition, and modification of fungal cell wall (Melloul et al., 2016). This antibiosis effect was variable according to *S. maltophilia* strains and their fitness. Most of the clinical and environmental strains were able to induce an antibiosis effect but this effect was dependent on the bacterial growth. A higher bacterial concentration was needed for the environmental *S. maltophilia* strains to induce antibiosis because these strains grew weaker at 37°C compared with clinical strains. However, not all strains were able to induce antibiosis. A clinical strain originated from horse trachea (Sm_2-1) was not able to make Af13073 develop highly branched hyphae, but was able to form strong biofilm at 37°C. A bacterial inoculum of 1000-fold higher failed to modify the phenotype of *A. fumigatus*. A significant increase in mortality of *G. mellonella* larvae was seen upon co-inoculation with *A. fumigatus* and *S. maltophilia*, but this effect is bacterial strain-dependent. A synergistic effect on mortality was observed in co-inoculation of human clinical *S. maltophilia* strain (Sm13637) with Af13073.

In the present study, the antibiosis was evaluated with different strains of *S. maltophilia* and *A. fumigatus* originated from human, animal and environmental. All of the tested *A. fumigatus* strains were able to form single and mixed biofilms with the reference strain Sm13637, and to produce ECM enclosing the fungal hyphae with or without the bacteria. The antibiosis effect of Sm13637 on *A. fumigatus* was observed on all the tested *A. fumigatus* strains and it did not seem to be fungal strain-dependent. Previous studies have already evaluated the potential fungal strain-dependence of antagonism effect of bacteria such as *P. aeruginosa* (Mowat et al., 2010). The study of Mowat et al. conducted on different clinical strains of *A. fumigatus* showed no difference when the strains were co-inoculated with *P. aeruginosa*. Ferreira et al. (2015) and Nazik et al. (2017) have assessed the inhibition of *A. fumigatus* strains (9 CF + 3 non-CF, and 12 CF + 12 non-CF strains, respectively), in the presence of *P. aeruginosa.* As in our study, the authors found that *P. aeruginosa* had a similar effect on the fungal morphology, irrespective of the studied *A. fumigatus* strains.

The different *S. maltophilia* strains used in the present study did not show the same effect on the tested fungi, i.e., *S. maltophilia* strains were not all able to induce highly branched fungal phenotype at 37°C after 24 h of culture and with a starter inoculum of 10^6^ bacteria/mL. The environmental strains (genogroup D) and the clinical strains originated from animals (genogroup 2) had weaker growth at 37°C in planktonic phase than the clinical strains of genogroup 6 (humans and animals). However, at the same temperature, Sm_2-1 of genogroup 2, isolated from horse, was able to form bulky biofilm (3 times bigger, similar to genogroup 6 strains) compared with other strains of genogroup 2 and D. The difference of *S. maltophilia* fitness at 37°C could explain the difference of their antibiosis effect which seems to be concentration-dependent. When a higher concentration of the bacteria (10^8^ cells/mL) from environmental strains was inoculated, fungal growth inhibition and modification of hyphal phenotype were observed in the mixed biofilm. A similar fungal growth inhibition was induced by Sm_2-1 at 10^8^ cells/mL in mixed biofilm with no modification of hyphal phenotype. Thus, the highly branched phenotype of *A. fumigatus* is attributed to bacterial growth (bacterial-concentration dependent) or to a deficiency in the putative production of the antifungal compound(s).

Several studies showed the effect of bacterial strain origins on fungal antagonisms, e.g., between *A. fumigatus* and *P. aeruginosa* (Ferreira et al., 2015; Shirazi et al., 2016). The authors observed a significant difference of fungal inhibition caused by *P. aeruginosa* isolates taken from CF-patients and non-CF patients; where the first had a stronger inhibition of *A. fumigatus* than the second. Some studies have demonstrated the antagonism interactions between *A. fumigatus* and *P. aeruginosa* via different kind of molecules, such as proteins, dirhamnolipids and phenazines (Yadav et al., 2005; Mowat et al., 2010; Briard et al., 2015, 2017; Shirazi et al., 2016; Sass et al., 2018), which the microorganisms secrete facing competition for the same nutrients and space. However, when the two microorganisms are separated by mass spectrometry. *P. aeruginosa* release volatile compounds that promote *A. fumigatus* growth and subsequently the invasion of the lung parenchyma (Briard et al., 2016).

Bacteria like *S. maltophilia* or *Lysobacter enzymogenes* produce antifungal metabolites such as maltophilin, dihydromaltophilin or heat-stable antifungal factor (HSAF), and polycyclic tetramate macrolactams (Jakobi et al., 1996; Yu et al., 2007; Su et al., 2017). Studies showed the antagonism triggered by the bacteria via those molecules on fungal species, such as *A. nidulans* (Li et al., 2006) or *Candida albicans* (Ding et al., 2016). Maltophilin, a macrocyclic lactam agent, was described as an antifungal agent several years ago and could be accounted for the antibiosis effect of *S. maltophilia* on *A. fumigatus* (Jakobi et al., 1996; Yu et al., 2007; Ding et al., 2016). Other factors, still not well characterized, can affect the mixed biofilm formation. Under this account, *S. maltophilia* biofilm can strongly be affected by temperature, pH and even by trimethoprim-sulfamethoxazole, as observed on a collection of different strains of *S. maltophilia* (Biocanin et al., 2017). Corlouer et al. (2017) reported that the *S. maltophilia* genogroups 6 and 2 were the most common strains in a French hospitalized population and they represented 75% of the strains isolated from patients with CF. These *S. maltophilia* strains seem to be well adapted to human and animal hosts because of their fitness at 37°C. Moreover, according to the present study, the pathogenicity of these strains might increase by the presence of *A. fumigatus*, as described in a recent study (Granchelli et al., 2018).

In alignment with the previous reports on *P. aeruginosa* and *A. fumigatus* (Reece et al., 2018), we described a significant increased in larvae mortality rate when *A. fumigatus* and *S. maltophilia* were inoculated in the same time, except for *S. maltophilia* of genogroup 2. This effect is dependent on the *S. maltophilia* strain used in the combination. Two *S. maltophilia* strains isolated from human clinical cases contributed to the increase larvae mortality in co-infection with the *A. fumigatus* strain originated from invasive aspergillosis patient. This *in vivo* effect do not seem correlated with the antibiosis effect observed *in vitro* at 37°C. This could also be explained by, for instance, the strain-dependent variation of maltophilin biosynthesis (Jakobi et al., 1996). The virulence of the other *S. maltophilia* strains was not synergistically increased in the presence of Af13073, except for one environmental strain. Interestingly, the clinical strain Sm_6-1 originated from CF-patient is the most virulent strain in *G. mellonella* model and incurred high mortality, suggesting that these isolates could be more pathogenic. It might be interesting to inoculate 2 strains of *S. maltophilia* and *A. fumigatus* from the same CF patient to analyze their virulence in *G. mellonella*, as described recently for *P. aeruginosa* and *A. fumigatus* (Reece et al., 2018). We probably would not have an increase in larvae mortality due to adaptation of these isolates to co-exist in the same environment.

Overall, these findings show specific bacterial and fungal interactions that could modify their pathogenicity in the respiratory tract of patients.

In summary, this is the first study to demonstrate that human, animal, and environmental strains of *A. fumigatus* and *S. maltophilia* form single and mixed biofilms. The latter are Sm strain-dependent, thus are variable in terms of thickness and antibiosis on *A. fumigatus*. It seems that the production of molecules that can modify the fungal phenotype is *S. maltophilia* strain dependent, e.g., some strains do not produce such molecules or produce very little. Moreover, bacterial clinical strains of genogroup 6 form a weaker mixed biofilm and a more highly branched *A. fumigatus* phenotype than the environmental strains, probably due to an adaptation to body temperature. In such conditions, *A. fumigatus* cell wall thickness increased which might be correlated with the modified antifungal susceptibility. However, it will be necessary to test a larger number of strains in order to assess the origin effect of *S. maltophilia* strains on mixed biofilm.

Further studies covering *A. fumigatus* resistance mechanisms, like efflux pumps modifications, in the presence of *S. maltophilia*, could be interesting in patients having respiratory tract chronic infections caused by these two microorganisms. Finally, the mixed biofilm model could be an interesting experimentation field to evaluate the susceptibility to relevant antimicrobial agents, alone and in combination.

## Author Contributions

EM and FB coordinated the design of the study. EM and LR participated in standardization of biofilms, qPCR, and crystal violet quantification, carried out TEM, and fluorescent microscopy. VR and DJ participated in *in vitro* experiments. M-FD performed the *in vivo* experiments in *G. mellonella* model. EM performed the statistical analysis. J-WD and P-LW performed the genotyping of *S. maltophilia* and helped with the analysis of the bacteria. FB and EM draft writing of the manuscript. LR, ED, JG, J-WD, and P-LW participated in the analysis of the results and have completed the manuscript. All authors read and approved the final manuscript.

## Conflict of Interest Statement

Over the past 5 years, FB has received grants from Astellas, payments for lectures from MSD, and travel expenses from Pfizer, MSD and Astellas. ED has received money for board membership from Astellas and Innothera, grants from Gilead, Ferrer, and Biorad, payments for lectures from Gilead and MSD, and travel expenses from MSD and Astellas. The remaining authors declare that the research was conducted in the absence of any commercial or financial relationships that could be construed as a potential conflict of interest.

## Footnotes

1 http://pubmlst.org/smaltophilia/

2 http://doua.prabi.fr/software/seaview

3 http://imagej.nih.gov/ij/
